# The Expression of the Beta Cell-Derived Autoimmune Ligand for the Killer Receptor Nkp46 Is Attenuated in Type 2 Diabetes

**DOI:** 10.1371/journal.pone.0074033

**Published:** 2013-08-29

**Authors:** Chamutal Gur, Jonatan Enk, Efraim Weitman, Etty Bachar, Yaron Suissa, Guy Cohen, Rachel Ben-Haroush Schyr, Helena Sabanay, Elad Horwitz, Benjamin Glaser, Yuval Dor, Ariel Pribluda, Jacob H. Hanna, Gill Leibowitz, Ofer Mandelboim

**Affiliations:** 1 The Lautenberg Center for General and Tumor Immunology, The Department of Immunology and Cancer Research, The Institute for Medical Research Israel Canada; and The Hebrew University-Hadassah Medical School, Jerusalem, Israel; 2 Department of Internal Medicine, Hadassah-Hebrew University Medical Center, Jerusalem Israel; 3 Department of Pathology and the Lautenberg Center for Immunology, Institute for Medical Research Israel-Canada, Hebrew University Hadassah Medical School, Jerusalem, Israel; 4 Endocrinology and Metabolism Service, Department of Internal Medicine, Hadassah-Hebrew University Medical Center, Jerusalem, Israel; 5 Department of Developmental Biology and Cancer Research, The institute for Medical Research Israel-Canada, The Hebrew University-Hadassah Medical School, Jerusalem, Israel; 6 Institute for Drug Research-Department of Pharmacology, The Hebrew University of Jerusalem, Jerusalem, Israel; 7 Electron Microscopy Center, The Weizmann Institute of Science, Rehovot, Israel; 8 Department of Molecular Genetics, The Weizmann Institute of Science, Rehovot, Israel; Tulane University, United States of America

## Abstract

NK cells rapidly kill tumor cells, virus infected cells and even self cells. This is mediated via killer receptors, among which NKp46 (NCR1 in mice) is prominent. We have recently demonstrated that in type 1 diabetes (T1D) NK cells accumulate in the diseased pancreas and that they manifest a hyporesponsive phenotype. In addition, we found that NKp46 recognizes an unknown ligand expressed by beta cells derived from humans and mice and that blocking of NKp46 activity prevented diabetes development. Here we investigated the properties of the unknown NKp46 ligand. We show that the NKp46 ligand is mainly located in insulin granules and that it is constitutively secreted. Following glucose stimulation the NKp46 ligand translocates to the cell membrane and its secretion decreases. We further demonstrate by using several modalities that the unknown NKp46 ligand is not insulin. Finally, we studied the expression of the NKp46 ligand in type 2 diabetes (T2D) using 3 different *in vivo* models and 2 species; mice and gerbils. We demonstrate that the expression of the NKp46 ligand is decreased in all models of T2D studied, suggesting that NKp46 is not involved in T2D.

## Introduction

NK cells are able to quickly kill virus-infected cells, tumors, bacteria, parasites and even self cells. The activity of NK cells is controlled by inhibitory and activating NK cell receptors [1]. The inhibitory receptors recognize mainly MHC class I proteins [2] and the activating receptors recognize various ligands which are tumor-derived, stress-induced, pathogen-derived or self-ligands [1]. Several killer receptors are expressed by NK cells, among which are NKG2D and the Natural Cytotoxicity Receptors (NCRs), which include 3 members: NKp30, NKp44 and NKp46 [1]. Mice express orthologous receptors for NKp46 (named NCR1) and for NKG2D [1], [3], [4]. While the ligands of NKG2D are well defined [5], [6], the identity of the NCR ligands, in particular those of NKp44 and NKp46, are still largely unknown. In this regard, it was shown that NKp44 and NKp46 recognize viral hemagglutinins [7], [8]. However both receptors also recognize unknown cellular ligands expressed by tumor cells, dendritic cells (DC) and stellate cells. In addition, NKp46/NCR1 also recognizes an unknown ligand expressed by pancreatic beta cells [1], [9], [10], [11], [12]. The beta cell ligand for NKp46 is expressed during early development, both in humans and mice [10], [11] and its expression is maintained, under conditions of beta cell regeneration and during the early stages of autoimmune diabetes [10]. Thus, discovering the identity of the NKp46 ligand expressed by beta cells is important as this could lead for the development of novel modalities for the treatment of T1D. Unfortunately, the identification of the cellular ligands for NKp46 has turned out to be a very difficult task and although the NKp46 and NCR1 receptors were discovered more than a decade ago the identity of their cellular ligands remains obscure.

Type 2 diabetes mellitus (T2D) results from failure of the pancreatic beta cells to cope with increased secretory demand induced by obesity and the associated insulin resistance. This might cause marked depletion of pancreatic insulin content [13], resulting in part from reduced beta cell mass [13]. The loss of beta cell mass occurs probably due to the noxious metabolic environment of T2D, including hyperglycemia and elevated free fatty acids, which leads to oxidative and ER stress. Inflammation has been implicated in the pathophysiology of both T1D and T2D [14]. In T2D, macrophages invade the islets and secrete IL-1β as well as other cytokines and chemokines, hence leading to beta cell dysfunction and apoptosis. However, unlike T1D, the islet inflammatory response of T2D is of "low-grade" and its role in the pathophysiology of T2D is somewhat controversial [14]. Finally, whereas T1D results from an autoimmune attack, which leads to near complete ablation of the beta cells, in T2D, beta cell loss is smaller. The differences in the phenotype and severity of the inflammatory response between T1D and T2D may suggest that certain components of the inflammatory response are dissimilar in the two forms of diabetes. Here we studied the properties of the beta cell ligand for NKp46 and the involvement of NKp46 in T2D.

## Materials and Methods

### Ethics statement

All murine studies were performed in a specific, pathogen-free unit of the Hadassah Medical School (Ein-Kerem, Jerusalem) in accordance with the guidelines of the ethical committee. The institutional ethics committee of the Hebrew University Hadassah Medical School approved this study. The ob/ob mice (B6.V-Lepob/J) and the db/db mice (BKS.Cg -+ Leprdb/+ Leprdb/OlaHsd) were obtained from the Jackson Laboratory and from the USA Harlan Laboratories, respectively. Psammomys obesus (*P. obesus*) were obtained from Israel Harlan Laboratories. *P. obesus* are out bred and the animals are not maintained under SPF conditions.

### Type 2 diabetes induction in *Psammomys obesus (P. obesus)*

Diabetes-prone male, 8–10 weeks of age *P. obesus* gerbils (150–200 g) were fed a low-energy (LE) diet (9.96 kJ/g; Koffolk, Petach-Tikva, Israel) to maintain normoglycemia (random non- fasted blood glucose <100 mg/dl). To induce diabetes (non-fasted blood glucose >200 mg/dl in two sequential measures), *P.obesus* were fed a high-energy (HE) diet (14.23 kJ/g; Teklad Global Diets, Boston, MA) for up to 24 days. To restore insulin secretion and normoglycemia, diabetic *P. obesus* fed a HE diet were fasted overnight, switched to a LE diet for 48 hours or treated by I.P injections of 1 nmol/kg Exendin (BACHEM cat. no.: H-8730, Lot 2500234) for 24 days.

### Fusion proteins, immunohistochemial and immunofluoroscense staining

All fusion proteins used in this study were generated in COS-7 cells and purified by affinity chromatography using a protein G column, as previously described [10], [11]. The immunohistochemial and immunofluoroscense stainings were performed as previously described [10], [11]. Staining was performed using fusion proteins containing the binding D2 domain of human NKp46 fused to human IgG1. This protein is named NKp46-Ig. As negative control we used either CD16-Ig or the KIR2DS4-Ig fusion proteins.

### Immunogold Electron Microscopy (EM)

For immunogold EM staining, cells were fixed with 0.254% glutaraldehyde and 3% paraformaldehyde solution, in cacodylate buffer containing 5 mM CaCl_2_ for 1 hour at 24°C. Fixed cell pellets were incubated in 10% gelatin at 37°C for 30 min, then centrifuged and excess of gelatin was removed at 37°C, followed by post-fixation in the same fixative at 4°C for 24 hours. Fixed cell pellets were cryoprotected by overnight infiltration with 2.3 M sucrose in cacodylate buffer then frozen by plunging into liquid nitrogen and ultrathin (75 nm) frozen sections were cut with a diamond knife at –120°C in a Leica EM FC6 ultramicrotome. The sections were transferred to formvar coated 200 mesh nickel grids. Sections were treated with conditioning medium for 5 minutes to block non-specific binding followed by 2 hour incubation with primary antibodies NKp46-Ig and the control KIR2DS4-Ig (concentration-0.048 µg/µl). After extensive washing in PBS-0.1% glycine, samples were incubated with polyclonal rabbit anti-human biotinylated secondary antibody and then with goat anti-rabbit (Jackson immunoresearch) 15 nm colloidal gold conjugate. For Insulin staining primary polyclonal guinea pig anti-mouse insulin (1:200, Abcam) and a secondary donkey anti-Guinea Pig 6 nm colloidal gold conjugate (Jackson immunoresearch) were used. Finally, the grids were washed in PBS-glycine stained with neutral uranyl acetate oxalate for 5-minutes and then with 2% uranyl acetate in H_2_O for 10 minutes. The grids were then embedded in 2% methyl cellulose/uranyl acetate and analyzed in a FEI T12 Spirit transmission EM; pictures were taken with an Eagle CCD-camera.

### Cells

The rat insulinoma cell line INS-1E [15] was kindly provided by Dr. M Walker, The Weizmann Institute of Science, Rehovot, Israel.

### FACS analysis

INS-1E cells were harvested, washed and stained using fusion proteins (3 µg/well) for 2h on ice. Following 2 washes the cells were stained with secondary alexafluor-647 conjugated donkey anti human-IgG (1:200, Jackson) for 30 min at room temperature.

### ELISA assays

To determine the interaction between insulin and the NKp46 receptor, plates were coated with capture anti human-IgG1 (1∶7500), followed by blocking in PBS 0.1% tween supplemented with 10% FCS. The plates were then incubated with medium containing various fusion proteins (1 µg/well). Next, the plates were washed and incubated with either biotynilated (bio-) recombinant insulin (0.2 µg/well, Sigma), with bio-anti-NKp46([7] 0.2 µg/well), used as a positive control or with bio-anti-CD3 (Abcam, 0.2 µg/well) used as a negative control. Binding was then detected by Streptavidin-HRP (Jackson ImmunoResearch, 1:1000) and the TMB reagent (SouthernBiotech, 200 µl/well).

To detect insulin secretion during glucose stimulation, we used Mercodia insulin ELISA kit (cat no 10-1247-01).

To detect the secreted NKp46 ligand plates were coated with 100 µl/well of various supernatants. After blocking with PBS-BSA 1% the indicated fusion proteins (0.1 µg/well) were added and incubated for 4 hours at room temperature (R.T). Plates were washed and polyclonal rabbit anti-human biotinylated antibody (1∶200 Jackson) was added and incubated for 1 hour at R.T. Streptavidin-HRP (Jackson ImmunoResearch, 1:1000) and TMB reagent (SouthernBiotech, 200 µl/well) were next added.

### BW reporter assay

The murine T cell line BW cells expressing NKp46 fused to the zeta chain were used [7]. The cells were incubated with insulin, glucagon or BSA and IL-2 secretion was detected using standard ELISA.

### Static incubations for insulin secretion measurements

INS-1E cells (10^5^/well) were pre-incubated for 1 hour in Krebs-Ringer bicarbonate (KRB) HEPES–BSA buffer containing 3.3 mM glucose at 37°C, followed by 1 hour incubation at 3.3 mM and additional 10 min and 30 min incubations in KRB-HEPES-BSA containing 16.7 mM glucose.

### Adeno-leptin preparation

The adeno-leptin vector was purchased from "vectorbiolabs". Additional viruses were produced by infection of GH374 cells. Virus titers were determined and mice were injected intravenously once.

## Results

### The expression of the NKp46 ligand is linked to insulin secretion

We have previously demonstrated that human and mouse beta cells express an unknown ligand for NKp46/NCR1 [10], [11]. While performing the experiments described in our previous publications we noticed that the expression of the NKp46 ligand tightly correlated with that of insulin. Therefore we thought to study the impact of insulin secretion on NKp46 ligand expression. Since the identity of the NKp46 ligand is unknown we used a fusion protein consisting of the extracellular binding domain of human NKp46 fused to human IgG1 to detect its expression.

**Figure 1:**
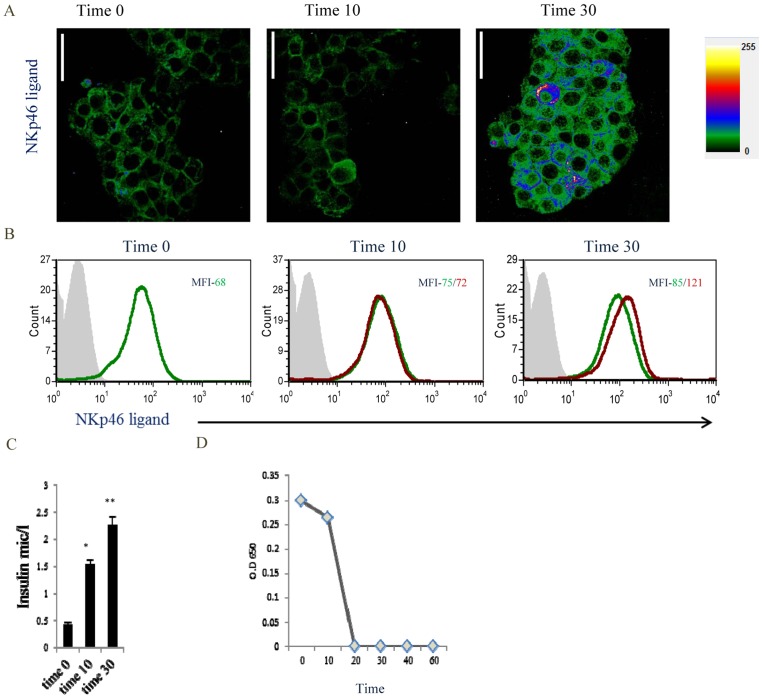
Stimulation of insulin secretion increases the expression of the NKp46 ligand. (A-B) INS-1E cells were incubated for different periods of time (indicated in the Figure) in Krebs-Ringer bicarbonate HEPES–BSA buffer containing 3.3 mM and 16.7 mM glucose. Cells were harvested at each time point and NKp46 ligand expression was analyzed by cytospin (A) and by flow cytometry (B). Shown in (A) is the intensity (colors intensity is indicated in the right of the Figure) of the expression of NKp46 ligand in INS-1E cells using the NKp46-Ig. MagnificationX400, scale-50 µm. (B) Flow cytometry staining for the surface expression of the NKp46 ligand in INS-1E cells following stimulation at 16.7 mM glucose (green histograms-unstimulated cells, red-stimulated). The MFI values are indicated in the corresponding histograms. (C) INS-1E cells shown in A were incubated at 16.7 mM glucose for different periods of time and secreted insulin was analyzed by an ELISA assay. Results are expressed as microgram/liter (mic/l). Error bars (SD) are derived from triplicates. Data from one of three independent assays is shown.*p = 0.0125 (0/10min), **p = 0.014 (10/30min). (D) ELISA assays for the detection of the NKp46 ligand in the supernatants of the INS-1E cells shown in (A) before and during glucose stimulation. Data from one of two independent assays is shown.

INS-1E beta cells were incubated in medium containing low glucose (3.3 mM) followed by glucose stimulation (16.7 mM) for different time periods. NKp46 ligand expression and localization were analyzed by confocal microscopy (Figure 1A) and by flow cytometry (Figure 1B). As expected, high glucose stimulated insulin secretion (1C) and this was associated with increased NKp46 ligand expression inside, and on the cell surface of INS-1E cells (Figure 1A and B). We next tested whether the NKp46 ligand is secreted in response to glucose stimulation. Interestingly, we found that the NKp46 ligand was constitutively secreted to the supernatant of unstimulated cells (time 0). Furthermore, we also observed that the NKp46 ligand is secreted during the initial steps of glucose-stimulated insulin secretion (time 10) (Figure 1D). However, during longer stimulation periods (time 20–60), concomitantly with the increased expression of the NKp46 ligand on the cell surface (Figure 1B), its secretion into the medium, disappeared (Figure 1D).

### The NKp46 ligand is not insulin

**Figure 2:**
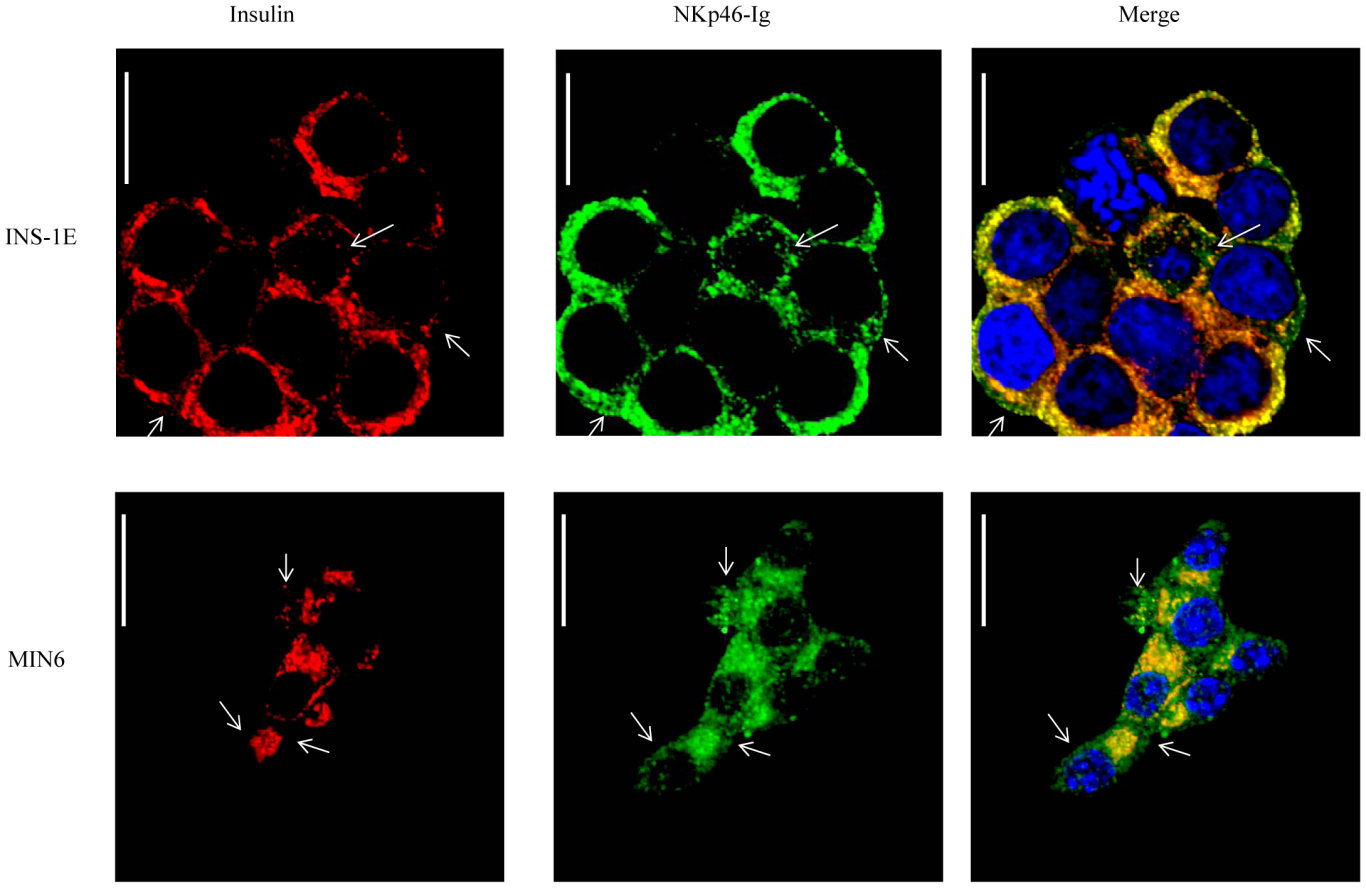
Insulinoma staining. (A-B) Immunofluorescence images of INS-1E (A) and MIN6 (B) beta cell lines. Left images represent staining with anti-insulin antibody (Insulin, red), middle images represent staining with the NKp46-Ig (NKp46 ligand, green) and the right images represent the merge signal (Merge, yellow). Arrows indicate areas positive for NKp46-Ig staining and negative for insulin. Representative of three independent staining is shown. Magnificationx1800, scale bar-10 µm.

To further characterize the regulation of NKp46 ligand expression we stained the insulinoma cell lines INS-1E and MIN6 with anti-insulin and with NKp46-Ig (Figure 2, red and green respectively). As can be seen in Figure 2, insulin co-locolized with NKp46-Ig in both cells, however, green dots (indicative for NKp46-Ig staining only, some are marked by arrows) were clearly seen in the merge pictures, suggesting that the NKp46 ligand is not insulin. No staining was observed when the control CD16-Ig fusion protein was used (Figure S1A).

**Figure 3:**
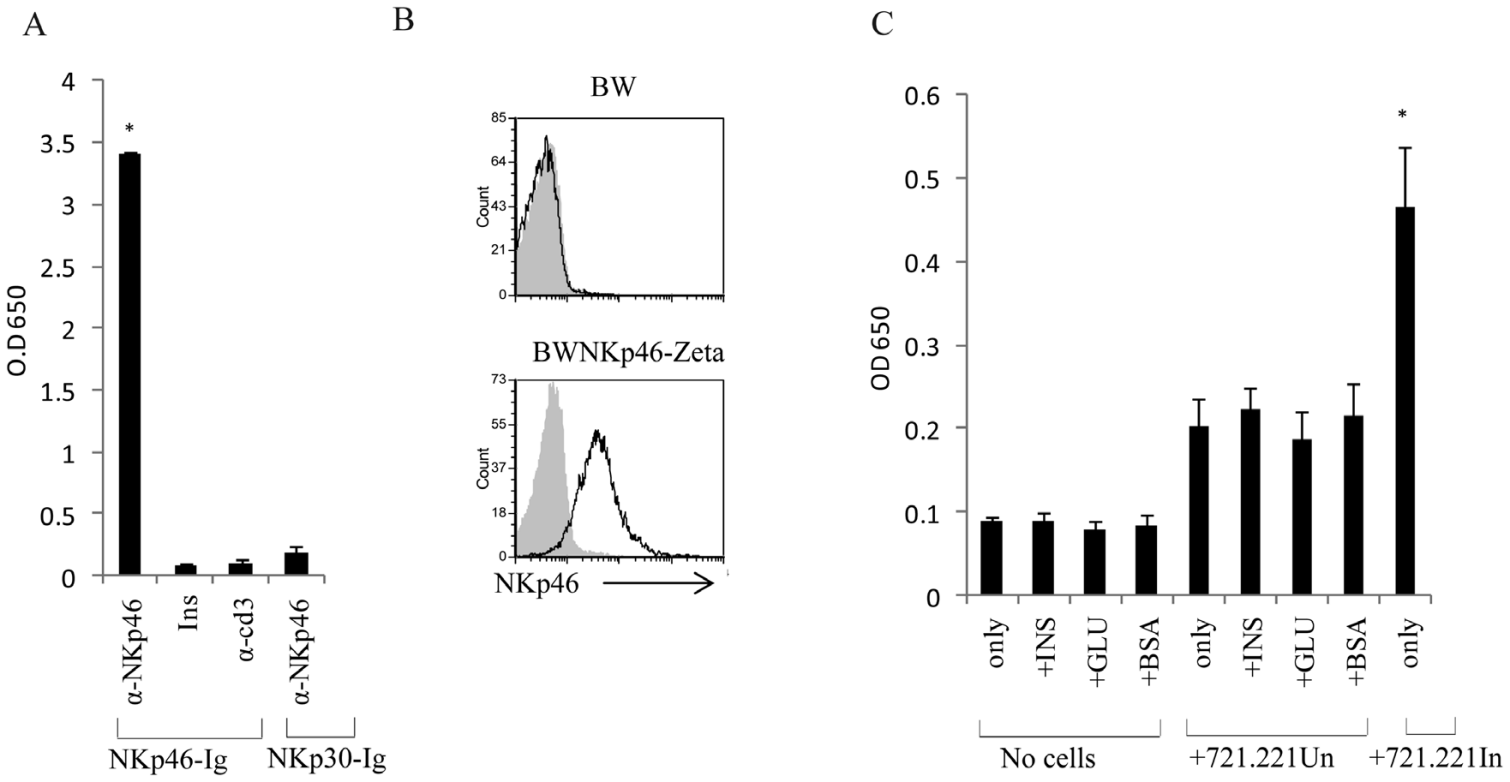
NKp46 does not bind to insulin. (A) Plates were coated with the indicated Ig-fusion proteins (NKp46-Ig and NKp30-Ig, X axis) and then incubated either with biotinylated antibodies (α-NKp46, and α-CD3) or with biotinylated insulin (Ins). ELISA detection was performed using Streptavidin-HRP. (B) FACS analysis for the expression of NKp46 on parental BW cells (top) and on NKp46-CD3-zeta (bottom) transfected BW cells. (C) NKp46-CD3-zeta transfected BW cells were incubated with insulin, glucagon or BSA, in the presence (+721.221Un) or in the absence of 721.221 cells. In addition, 721.221 cells infected with PR8 influenza (+721.221In) were incubated with NKp46-CD3-zeta transfected BW cells. IL-2 was detected in the supernatants using standard ELISA.

To directly demonstrate that insulin is not the NKp46 ligand, we used several experimental approaches. First, an ELISA assay was conducted in which plates coated with capture anti human-IgG1 were incubated with NKp46-Ig, or with NKp30-Ig fusion proteins. The plates were then incubated with either biotynilated-recombinant insulin, with anti-NKp46, as a positive control or with anti-CD3 (α CD3) as a negative control. NKp30-Ig incubated with anti-NKp46 mAb was used as an additional negative control. As can be seen in Figure 3A, the fusion proteins were indeed bound by the capture antibodies, as the NKp46-Ig was detected by the anti-NKp46 mAb. However, insulin did not bind to the fusion proteins, further suggesting that insulin is not the NKp46 ligand.

Another modality used to test whether insulin is a ligand for NKp46, was the BW reporter assay that was developed in our laboratory [16]. The murine T cell line BW was transduced with a chimeric protein consisting of the extracellular portion of NKp46 fused to the mouse zeta chain of CD3 (Figure 3B). Triggering of this reporter system by a ligand for NKp46 should lead to IL-2 secretion. To test whether NKp46 interacts with insulin we incubated the BW-NKp46 cells with purified insulin, BSA or glucagon in the presence or absence of the tumor cell line 721.221 that express an unknown ligand for NKp46 [7], or with 721.221 cells infected with influenza and expressing as a result hemagglutinin, a known ligand for NKp46 [7]. Insulin failed to induce IL-2 secretion from the BW-NKp46 cells under all conditions (Figure 3C). In contrast, incubation of BW NKp46 cells with 721.221 cells lead to increased IL-2 secretion, which was further enhanced following incubation with the influenza-infected 721.221 cells (721.221In, Figure 3C). Collectively, our findings indicate that insulin is not a ligand for NKp46.

**Figure 4:**
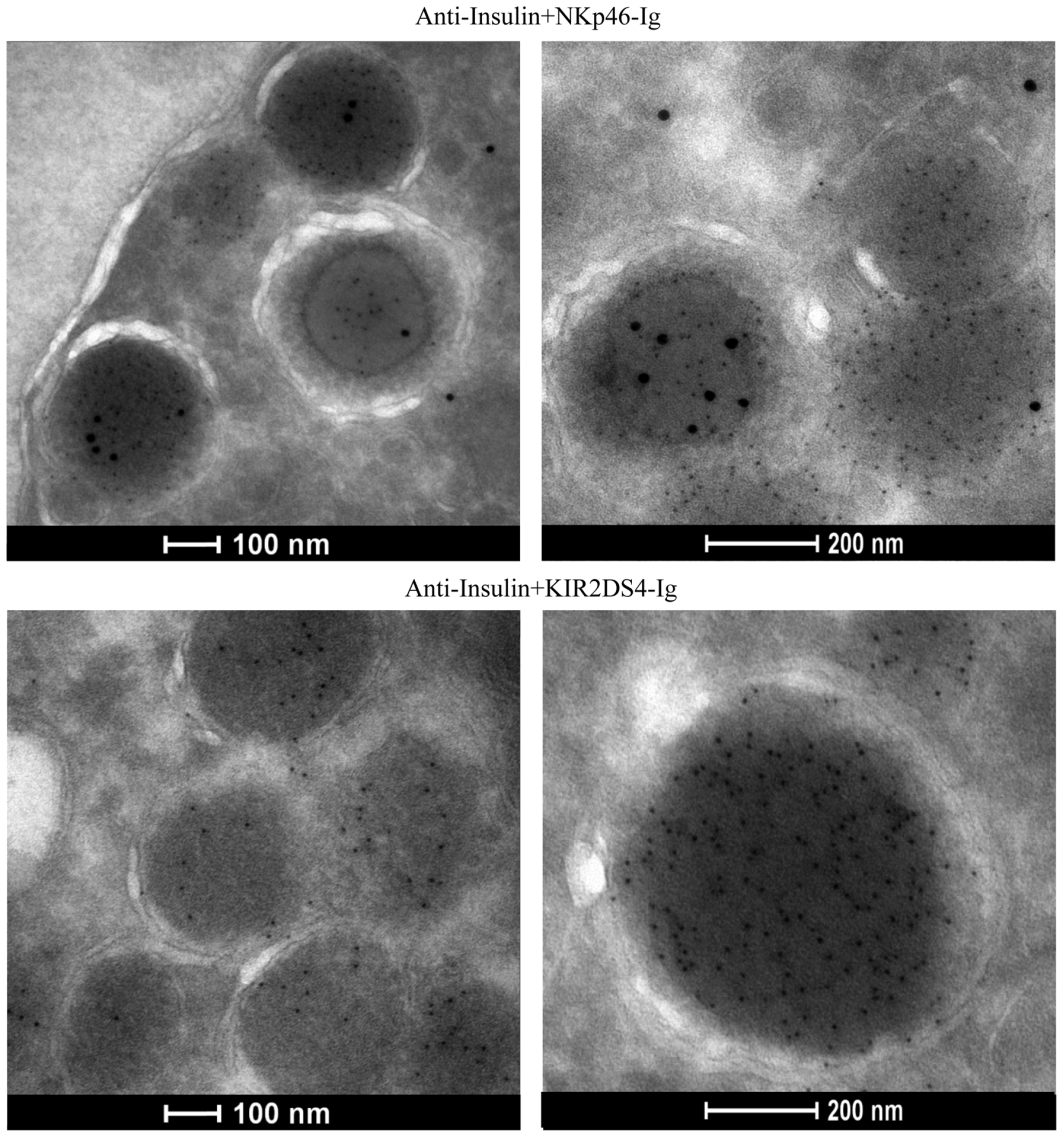
Electron microscopy images of NKp46 ligand and of insulin. INS-1E cells were co-stained with NKp46-Ig (upper, large black dots, 15 nm) and KIR2DS4-Ig (lower) together with anti-insulin (small black dots, 6 nm).

To gain more insight about the properties of the NKp46 ligand and to observe whether the NKp46 ligand is co-localized with insulin we co-stained INS-1E cells with NKp46-Ig or KIR2DS4-Ig (used as control), together with anti-insulin followed by Electron microscopy (EM) analysis. Importantly, we found that the NKp46 ligand is located mostly inside but also outside of the insulin secretory granules (large black dots, Figure 4). Furthermore, insulin that is present in secretory granules (indicated by the small dots) did not overlap with the NKp46 ligand (Figure 4, upper pictures), further indicating that insulin is not the beta cell ligand for NKp46.

### Reduced expression of insulin and NKp46 ligand/s in db/db and ob/ob mice

**Figure 5:**
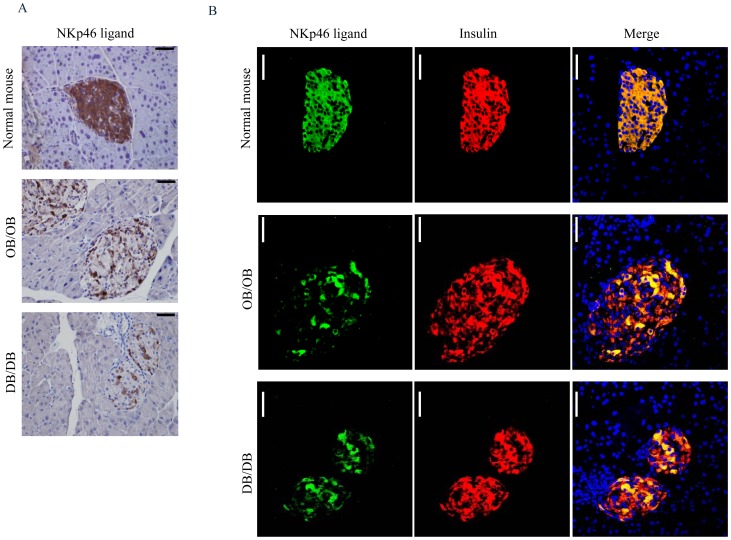
Expression of insulin and NKp46 ligand in T2D models. (A) Immunohistochemical staining of pancreatic tissues derived from 8–10 weeks old control C57BL/6 mice (top), 8–10 weeks old leptin deficient (OB/OB) mice (middle) and 5–6 weeks old leptin receptor deficient (DB/DB) mice (bottom). Staining was performed with NKp46-Ig. Magnificationx400. Representative of three independent staining is shown. Tissues were obtained from 3–4 mice in each group. (B) Immunofluorescence staining of pancreatic tissues derived from 8–10 weeks old control C57BL/6 mice (top), 8–10 weeks old OB/OB mice (middle) and 5–6 weeks old DB/DB mice (Bottom). Left images represent staining with NKp46-Ig (NKp46 ligand, green), middle images represent staining with anti-insulin (Insulin, red) and the right images represent the merge signal (Merge, yellow). Magnificationx400. Scale bars-50 µm. Representative of three independent staining is shown, 3–4 mice in each group.

We next studied whether NKp46 might play a role in T2D using two models of T2D; the leptin-deficient ob/ob mice and the leptin receptor-deficient db/db mice. Interestingly, immunohistochemical staining showed a marked decrease in the expression of the NKp46 ligand in both models (Figure 5A). This result was quite remarkable, as we have never observed reduced NKp46-Ig staining in pancreatic sections derived from normal mice or from pre-diabetic NOD mice or in pancreatic cells derived from humans [10], [11]. To further corroborate these observations, we performed co-immunofluorescence staining with NKp46-Ig and with anti-insulin. In both ob/ob and db/db mice, a patchy NKp46-Ig staining were observed, as opposed to the uniform NKp46-Ig staining observed in control islets (Figure 5B). Notably, not all insulin producing cells were stained by NKp46-Ig (Figure 5B). No staining was detected when a control-Ig fusion protein was used (Figure S1B). To ascertain that the patchy staining of the NKp46-Ig, observed in db/db and ob/ob mice is not due to deformation or destruction of the islet structure, islets were stained for glucagon (Figure. S1C) to identify the alpha cells located at the periphery of the islets, thus defining their borders. As can be seen, the architecture of the islets was preserved with glucagon-positive cells present in the mantle of the islets. Furthermore, the glucagon staining did not overlap with NKp46-Ig staining (Figure S1C), demonstrating that the expression of the unknown NKp46 ligand in the islets is restricted to beta cells.

### Leptin administration restores NKp46 ligand expression

**Figure 6:**
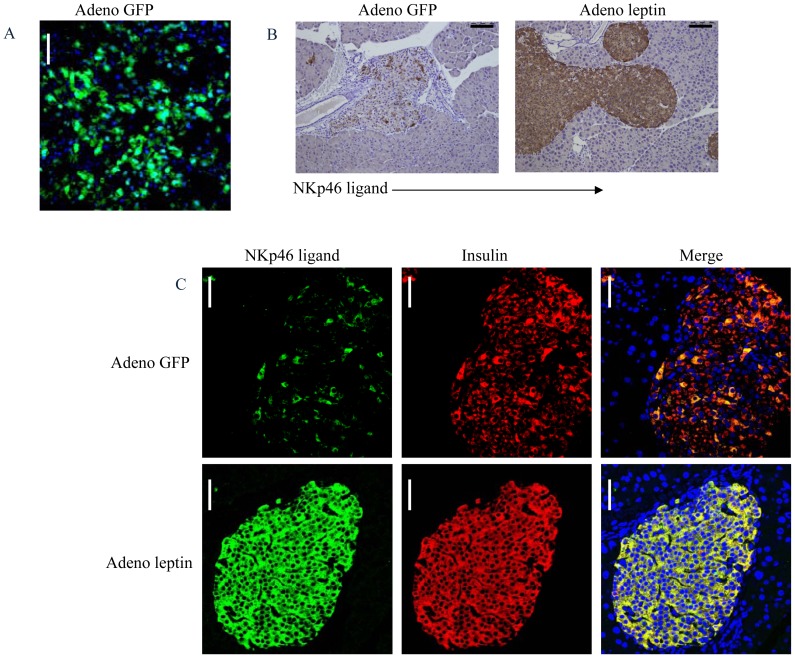
Recovery of NKp46 ligand after reconstitution of leptin in ob/ob mice. Mice (ob/ob) were injected I.V with an adeno-vector encoding for GFP (Adeno GFP, A-C) or with an adeno-vector encoding for murine leptin (B-C). (B-C) Immunohistochemical (B) and Immunofluorescence (C) NKp46-Ig staining of pancreatic tissue derived from 8–12 weeks male ob/ob mice treated for 14 days with adeno-vector encoding for leptin (B, right and C lower) or with adeno-vector encoding for GFP (B, left and C upper). (C) Left images represent staining with NKp46-Ig (NKp46 ligand, green), middle images represent staining with anti-insulin antibody (Insulin, red) and the right images represent merge signal (Merge, yellow). (A and C) Magnificationx400, scale bar-50 µm, (B) Magnificationx200, scale bar-25 µm. Representative of two independent experiments, 3–4 mice in each group.

We next studied whether reversal of T2D can restore the NKp46 ligand expression by using the ob/ob mice. For this purpose, we injected 8–12 weeks-old ob/ob mice with adeno-virus vectors expressing either leptin or GFP (used as a control). Prior to the injection, the ob/ob mice were obese (average initial body weight 53 g and 51 g in the leptin treatment and control groups, respectively), without prominent hyperglycemia. Fed blood glucose levels before injections were below 200 mg/dl in both groups. We initially confirmed that the liver was effectively transduced, as GFP expression (appeared as early as 1 day after infection) was detected in the livers of animals infected with the GFP control virus (Figure 6A).

The ob/ob mice started to lose weight already around one day post adeno-leptin infection. At 14 days following adeno-leptin infection, the ob/ob mice lost around 50% of their initial weight, reaching almost the weight of healthy mice (final average weight was 29 g, data not shown). At this stage, the islets of the adeno-leptin infected mice were still hypertrophic, however, strikingly, the expression of NKp46 ligand was restored (Figure 6B). The restoration of the NKp46 ligand expression following leptin treatment was associated with increased insulin expression (Figure 6C). These findings indicate that the changes in the expression of the NKp46 ligand that occur during the development of T2D are reversible.

### Expression of NKp46 ligand in *P. obesus*

**Figure 7:**
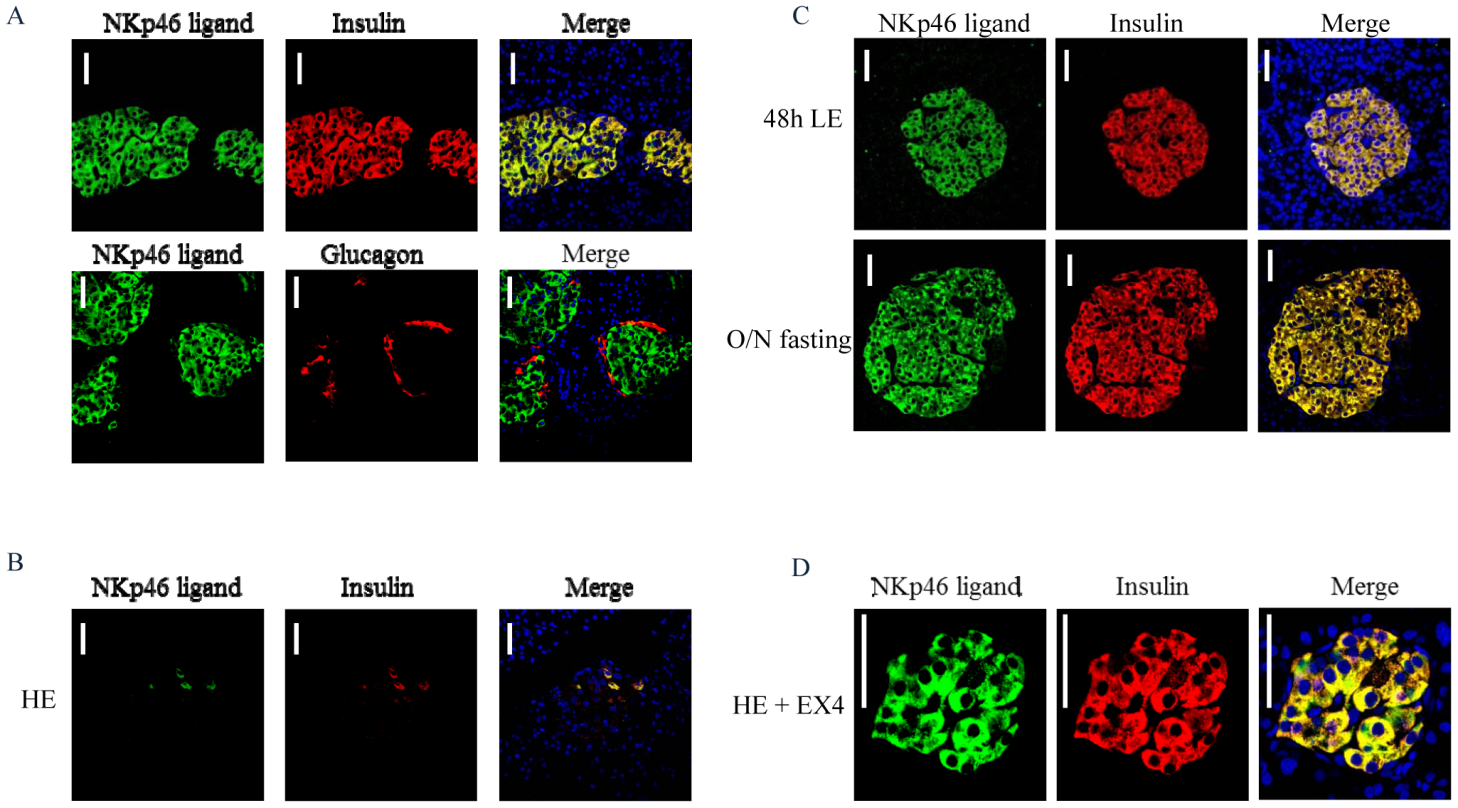
Expression of the NKp46 ligand in beta cells derived from *P. Obesus*. (A)Immunofluorescence staining of pancreatic tissues derived from 8–10 weeks of age, male, *P. obesus* gerbils fed with LE diet. In the upper images staining for insulin and for NKp46 ligand are shown and in the lower images staining for NKp46 ligand and glucagon are shown. (B) NKp46-Ig and anti-insulin staining of pancreases derived from diabetic (non-fasting blood glucose>250 mg/dl for 2 sequential days) *P. obesus* gerbils fed with HE diet for 21–24 days. (C) NKp46-Ig and anti-insulin staining of pancreatic tissues derived from diabetic *P. obesus* gerbils fed with HE diet for 21–24 days followed by 48 hours of LE diet (C, upper) or overnight (O/N) fasting (C, lower). (D) NKp46-Ig and anti-insulin staining of pancreatic tissues derived from non-diabetic *P. obesus* fed for 21–24 days with HE diet and then injected with Exendin 4 (EX4). For all Figure parts the left images represent staining with NKp46-Ig (NKp46 ligand, green), the middle images represent staining with anti-insulin (A, upper, B-D) or anti-glucagon antibodies (A, lower) and the right images represent the merge signal (Merge, yellow). (A-C) Magnificationx400. (D) Magnificationx1200. Scale bars-50 µm. Representative staining of pancreatic tissues derived from 2–4 animals per each group is shown.

*P. obesus* is a gerbil habituating in African and Mediterranean semi-desert regions [17], [18]. In their native habitat these gerbils feed on low calorie *Atriplex halimus* plants and are normoglycemic [17], [18], [19]. However, in captivity, under conditions of restrained physical activity and of high energy diet (HE) feeding, *P. obesus* rapidly develop hyperglycemia, hyperinsulinemia and marked depletion of pancreatic insulin content [17], [18], [19]. To test whether the expression of NKp46 is altered in the nutrition-induced diabetes of *P. obesus* we first performed co-immunofluorescence staining of pancreatic tissues derived from normoglycemic *P. obesus* fed a low energy (LE) diet. Consistent with our previous findings in mice and humans [10], [11], the beta cells of *P. obesus* also expressed the NKp46 ligand, whereas the alpha cells stained negative with NKp46-Ig (Figure 7A). Control staining is shown in Figure S1B.

*P. obesus* developed diabetes within a few days of feeding on an HE diet. Remarkably, after 21–24 days, the expression of both insulin and the NKp46 ligand almost completely disappeared (Figure 7B). Subsequently, diabetic *P. obesus* were switched to an LE diet for 48 h or fasted overnight blood glucose and this resulted in normalization of blood glucose levels and the restoration of both insulin and NKp46 ligand expression (Figure 7C). Finally, we treated diabetic *P. obesus* with the GLP-1 analog, Exendin 4, commonly used for the treatment of T2D [20]. Exendin 4 reversed diabetes and increased insulin content together with restoration of NKp46 ligand expression (Figure 7D).

## Discussion

Two NK killer receptors were shown to play a role in the pathophysiology of T1D diabetes; NKG2D and NKp46 [10], [11], [21]. These two receptors regulate T1D development in different ways as while the expression of NKG2D is not restricted only to NK cells [5], the expression of NKp46 is mainly NK cell-specific [1], [3], [22]. Furthermore, the beta cell recognition by these two receptors is also different. The NKG2D ligands are stress-induced, their identity is known and their expression can be detected only on beta cells derived from NOD mice [21]. In contrast, the identity of the beta cell ligand/s for NKp46 is unknown and the expression of this ligand is not restricted to the NOD mice, as healthy beta cells derived from mice, humans [10], [11] and as we demonstrated here, also from *P. obesus* express an unknown NKp46 ligand, indicating that this beta cell ligand is conserved among different species.

Identifying the NKp46 ligand may have important implications for T1D and possibly for other autoimmune disorders. Hitherto, the identity of the NKp46 ligand is still unknown. We partially characterized the biochemical properties of the ligand and found that it is degraded by proteases (trypsin and proteinase K) and that its binding to NKp46 depends on glycosylation of the receptor [10]. Here we show that the NKp46 ligand is located mainly inside insulin granules and that at low glucose it is being secreted. It is not clear whether the ligand is secreted by secretory granules along with insulin or through other yet undefined mechanisms. At high glucose, the NKp46 ligand is delivered to the plasma membrane, most probably by secretory granules containing NKp46 ligand that are transported to the membrane during exocytosis. Intriguingly, this was associated with disappearance of the ligand from the supernatants of the stimulated cells. This might be explained by the fact that upon glucose stimulation, insulin is released from newly formed insulin granules [23], that perhaps do not contain the NKp46 ligands. Moreover, it appears that the NKp46 ligand has a short half-life when secreted, as it disappeared completely and could not be found in the supernatant after incubation for more than 20 minutes.

Additionally, we showed that the NKp46 ligand is not insulin as the co-localization of the NKp46 ligand and insulin was partial. Furthermore, extra-granular expression of the NKp46 ligand was observed by EM. Finally, insulin did not bind and failed to activate NKp46.

We further show that in T2D, depletion of insulin content is associated with marked depletion of NKp46 ligand in the beta cells. This implies that insulin depletion in diabetic beta cells may protect them from NK cell attack and thus should be viewed as a protective response, preventing NKp46-mediated beta cell destruction under conditions of low-grade inflammation in T2D. Further support for this conclusion is given by studies showing decreased NK cell numbers and impaired NK cell function in leptin-deficient mice [24], suggesting that NK cells are not involved in the pathophysiology of T2D.

Finally, our findings have important clinical implications. The realization that the expression of the NKp46 ligand is associated with insulin implies that it might be possible to manipulate the expression of the ligand in the plasma membrane by inhibiting insulin secretion, which may render the beta cells less susceptible to an autoimmune attack. Indeed, treatment with the K+ ATP channel opener, diazoxide, which inhibits insulin secretion, protected beta cells and improved glycemic control in T1D [25]. Similarly, intensive insulin treatment, which is expected to inhibit endogenous insulin secretion, resulted in better preservation of beta cell function in T1D [26], [27]. Future studies will show whether these manipulations protect beta cells in T1D through modulation of NKp46 activity.

## Supporting Information

Figure S1 **Control staining.** (A) INS1E cells (top) and MIN6 cells (bottom) stained with control-Ig fusion protein (CD16-Ig, green), with anti-insulin (red) and with DAPI (blue). (B) Control staining of pancreatic islets derived from OB/OB (top), from DB/DB (middle) and from *P. obesus* (bottom) stained with a control fusion protein (CD16-Ig, green) and with anti-insulin (red). (C) Pancreatic islets from an OB/OB mouse (top) and DB/DB mouse (Bottom) stained with anti-glucagon (red) and NKp46-Ig (green). (A) Magnificationx1800, scale bar-10 µm. (B-C) Magnificationx400, scale bar-50 µm
(TIF)
