# Supplementary figures and images for: The Expression of the Beta Cell-Derived Autoimmune Ligand for the Killer Receptor Nkp46 Is Attenuated in Type 2 Diabetes
Source: PLoS One. 2013 Aug 29;8(8):e74033. doi: 10.1371/journal.pone.0074033 (PMC3757008; doi:10.1371/journal.pone.0074033)

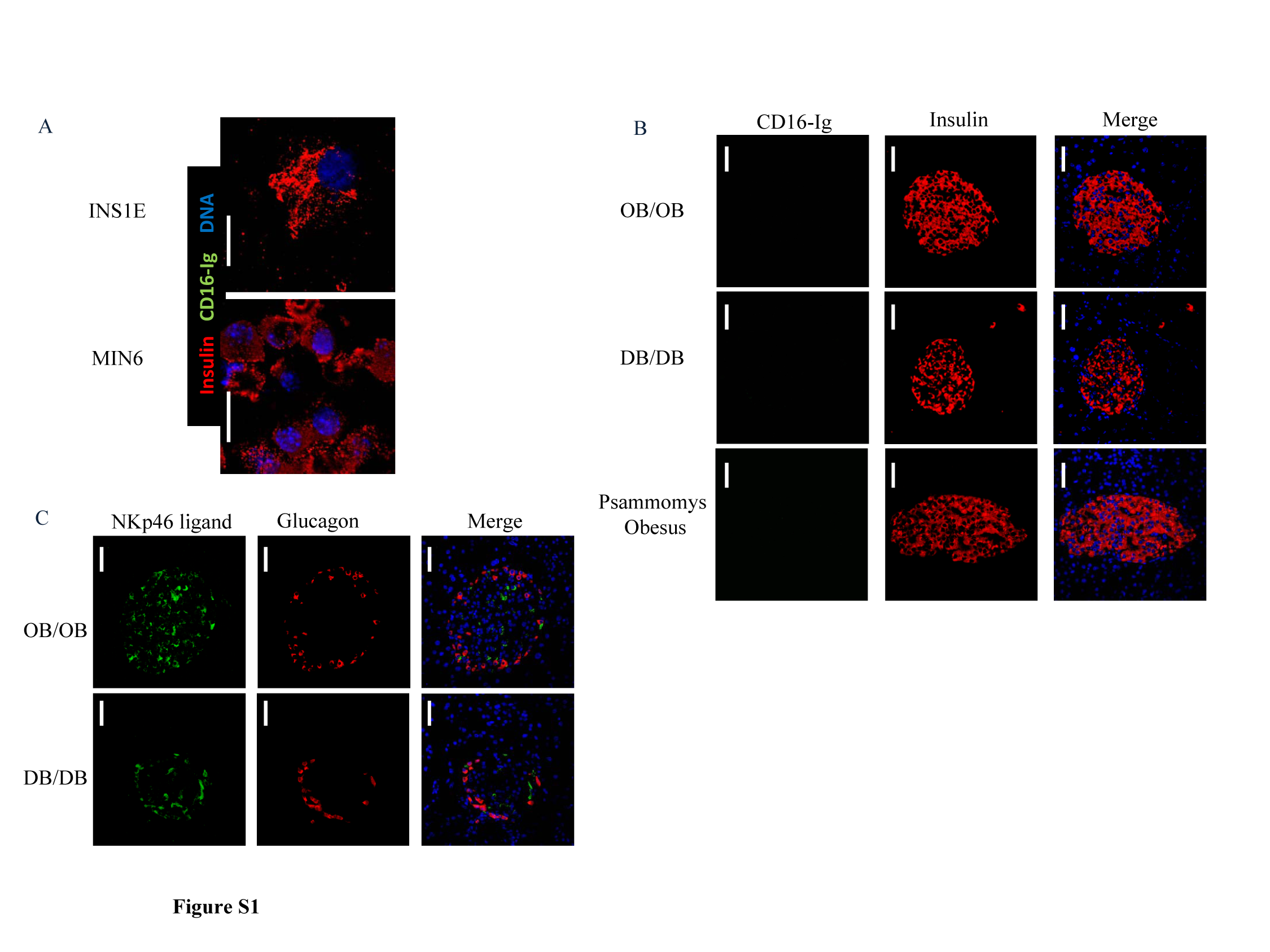

Supplement: Figure S1 — Control staining. (A) INS1E cells (top) and MIN6 cells (bottom) stained with control-Ig fusion protein (CD16-Ig, green), with anti-insulin (red) and with DAPI (blue). (B) Control staining of pancreatic islets derived from OB/OB (top), from DB/DB (middle) and from P. obesus (bottom) stained with a control fusion protein (CD16-Ig, green) and with anti-insulin (red). (C) Pancreatic islets from an OB/OB mouse (top) and DB/DB mouse (Bottom) stained with anti-glucagon (red) and NKp46-Ig (green). (A) Magnificationx1800, scale bar-10 µm. (B-C) Magnificationx400, scale bar-50 µm (TIF) [file pone.0074033.s001.tif]
